# Enhanced Photosynthetic Capacity, Osmotic Adjustment and Antioxidant Defenses Contribute to Improve Tolerance to Moderate Water Deficit and Recovery of Triploid Citrus Genotypes

**DOI:** 10.3390/antiox11030562

**Published:** 2022-03-16

**Authors:** Radia Lourkisti, Yann Froelicher, Raphaël Morillon, Liliane Berti, Jérémie Santini

**Affiliations:** 1 Laboratoire de Biochimie et Biologie Moléculaire du Végétal, Centre National de la Recherche Scientifique (CNRS), Unité Mixte de Recherche (UMR), 6134 Sciences pour l’Environnement (SPE), Université de Corse, 20250 Corte, France; berti_l@univ-corse.fr (L.B.); santini_j@univ-corse.fr (J.S.); 2 Centre de Coopération Internationale en Recherche Agronomique pour le Développement (CIRAD), UMR AGAP Institut, INRAE, Institut Agro, University Montpellier, 34398 Montpellier, France; yann.froelicher@inrae.fr (Y.F.); raphael.morillon@cirad.fr (R.M.); 3 CIRAD, UMR AGAP, 20230 San Giuliano, France; 4 CIRAD, UMR AGAP Institut, 34398 Montpellier, France

**Keywords:** antioxidant system, ROS accumulation, osmolytes, enzymatic antioxidant, *Citrus*, polyploidy, drought stress

## Abstract

Currently, drought stress is a major issue for crop productivity, and future climate models predict a rise in frequency and severity of drought episodes. Polyploidy has been related to improved tolerance of plants to environmental stresses. In Citrus breeding programs, the use of triploidy is an effective way to produce seedless fruits, one of the greatest consumer expectations. The current study used physiological and biochemical parameters to assess the differential responses to moderate water deficit of 3x genotypes compared to 2x genotypes belonging to the same hybridization. Both parents, the mandarin Fortune and Ellendale tangor, were also included in the experimental design, while the 2x common clementine tree was used as reference. Water deficit affects leaf water status, as well as physiological and detoxification processes. Triploid genotypes showed a better ability to maintain water status through increased proline content and photosynthetic capacity. Moreover, less oxidative damage was associated with stronger antioxidant defenses in triploid genotypes. We also found that triploidy improved the recovery capacity after a water deficit episode.

## 1. Introduction

Drought is considered the single most devastating environmental stress, limiting crop productivity more than any other form of environmental stress [1]. Climate models have predicted increased severity and frequency of drought under the ongoing global climate change scenarios [2,3]. Water deficit induces physiological changes in plants, such as stomatal closure, one of the first physiological responses. Stomatal closure is an effective way to decline water loss through transpiration but also reduces CO_2_ and nutrient uptake, impairing metabolic pathways [4]. Continued photosynthetic light reactions during water deficit under restricted CO_2_ availability results in the accumulation of reduced photosynthetic electron transport components, which can lead to over-production of reactive oxygen species (ROS). These species resulted in the reduction of oxygen and include free radicals such as superoxide anion (O_2_^•−^) or hydroxyl radical (HO^•^) and non-radical forms such as hydrogen peroxide (H_2_O_2_) [5]. Excessive ROS generation can cause severe cell damage, especially oxidation of proteins, lipids, and nucleic acids [6]. Among these damages, lipid peroxidation of biological membranes causes further damage of the photosynthetic apparatus through the decline of the membrane integrity. The varieties of secondary products, such as lipid hydroperoxides and various aldehydes generated during lipid peroxidation can be used as oxidative stress biomarkers [7]. For instance, malondialdehyde, one of the major aldehyde species produced from lipid peroxidation, has been widely used as biomarker of abiotic stress tolerance [8,9,10].

Plants have developed various complex mechanisms to cope with water deficit effects including decline in cell turgor, development of photo-inhibition, and oxidative stress. Osmotic adjustment (OA) helps in sustaining cell turgor through accumulation of compatible solute including sugars, inorganic ions, and amino acid when the leaf water potential is reduced [11]. Proline is known as the predominant compatible solute that accumulate under water deficit conditions [12]. Moreover, proline accumulation helps to cope effects of water deficit not only through OA, but also by detoxification of hydroxyl radical, protecting membrane integrity [13]. Thermal dissipation, also known as non-photochemical quenching (NPQ), is the main photo-protective mechanism for dissipating the majority of excess energy during drought [4]. This mechanism is mediated by the de-epoxidation of xanthophyll cycle, also involved in antioxidant defences [14]. The antioxidant defence system is essential to limiting ROS accumulation and thus maintaining cell redox homeostasis, and involves enzymatic and non-enzymatic compounds [15]. Enzymatic systems include superoxide dismutase (SOD, EC. 1.15.1.1), which acts as the first protection pathway by catalysing the superoxide anion dismutation to H_2_O_2_. Resulting H_2_O_2_ can be further detoxify by catalase (CAT, EC. 1.11.1.6) in peroxisome or ascorbate peroxidase (APX, EC. 1.11.1.11), which is ubiquitous in the plant cell [16] and involved in the ascorbate-glutathione (Asa-gsh) pathway. Ascorbate is the most significant antioxidant molecule in plant tissue and can directly scavenge ROS in addition with its role in Asa-gsh cycle [17]. Stronger antioxidant mechanisms have widely been associated with enhanced tolerance to abiotic stresses including drought stress [18,19]. Therefore, breeding drought tolerant genotypes may reduce drought effects and thus improve crop productivity.

Polyploidy is often related to a better tolerance to environmental stresses [20,21,22] thanks to genetic, metabolic, and cytological characteristics of polyploids [23]. Both enhanced photosynthesis and fruit metabolism were also observed in polyploids [24]. In the *Citrus* genus, most of species are diploid (2n = 2x = 18), but spontaneous triploid can be found among citrus cultivars such as in ‘Tahiti’ lime (2n = 3x = 27). Use of triploidy has been proven to be an effective breeding strategy to produce seedless cultivars and thus may improve the citrus fruit market [25,26]. Moreover, triploids are heterozygous, and each hybrid exhibits a specific allelic combination that can lead to high gene dosage and thus may contribute to better adaptation to stressful conditions [27]. While tetraploidy in citrus, especially in rootstocks, has been related to improved tolerance to abiotic stresses [28,29,30], the enhanced tolerance to biotic and abiotic stresses of triploid citrus scions remains to be confirmed [31,32]. 

In this study, we aimed to highlight the response to moderate water deficit of 3x citrus genotypes, compared to 2x, resulting from the hybridization between Fortune mandarin and Ellendale tangor performed by the INRAE-CIRAD research center (San Giuliano, Corsica, France) [33]. Both parents and 2x common clementine tree were also selected for this study. The response to water deficit and the recovery capacity of 3x genotypes was compared to 2x in terms of (i) leaf water status, (ii) physiological behavior (net photosynthesis, stomatal conductance, transpiration, Fv/Fm ratio and non-photochemical quenching), and (iii) cell oxidative markers and antioxidant defenses (antioxidant compounds and enzymes involved in detoxification processes).

## 2. Materials and Methods

### 2.1. Plant Material and Growth Conditions 

Diploid (2x) and triploid (3x) hybrid citrus trees from Fortune mandarin (*Citrus reticulate Blanco*) and Ellendale tangor (*Citrus reticulata* *Blanco × Citrus sinensis* (L.) Osb.) hybridization were used to perform this study. Both parents and 2x common clementine were also selected for the study. The 2x and 3x genotypes were selected among a population of 80 genotypes (40 diploids and 40 triploids) according their MDA content in leaves sampled in September 2017, as described in a previous study [34]. Diploid and 3x genotypes with the greatest MDA level (D1-2x, T1-3x and T2-3x) and others with the lowest level (D40-2x, T38-3x, T39-3x, and T40-3x) were selected for this study. All scions were grafted onto C-35 Citrange (*Citrus sinensis* *‘Ruby Blood’ × Poncirus trifoliata*) and grown in a greenhouse for seven months at the INRAE-CIRAD experimental station in San Giuliano, Corsica, France (42°17′07.5″ N, 9°31′21.9″ E). The plants were then transferred in 10 L pots with a substrate of sand, topsoil, and peat Klasmann TS1 (1:1:2) and grown in controlled conditions (27–30 °C day/20–25 °C night at 60% to 80% relative humidity). The same weight of soil was provided for each pot. Before the beginning of the stress, plants were watered near pot capacity and fertilized with nutritive solution. 

### 2.2. Water Deficit Treatment

Before water deficit treatment, plants were pruned to equalize tree size. To achieve the maximum water holding capacity, plants were irrigated to full saturation, and the excessive water was then drained off overnight. The pots were weighed to determine the weight at pot capacity. To prevent water loss, each pot was enclosed in a plastic bag loss. The experiment was performed in completely two randomized block design: 

The control group: 30 plants (three replicates per genotype) were maintained at pot capacity by daily watering.

The water deficit group: 30 plants (three replicates per genotype) were exposed to water deficit at soil water content of 45% and 55% of pot capacity. The watering was performed in each pot when the assigned minimal soil water content was reached. Each pot was weighed before and after watering to check the water conditions. The water deficit conditions were maintained for six days. At this time, the physiological parameters were recorded. After the water deficit treatment, stressed plants were re-watered (recovery period) with daily irrigation near pot capacity for five days to analyze the recovery capacity of the plants. Three independent biological leaf sample replicates were harvested from each genotype and treatment (control, water deficit and recovery). Leaves samples were further frozen and ground in liquid nitrogen and stored at −80 °C for biochemical assays. 

### 2.3. Drought-Related Parameters 

At the beginning and the end of the experiment, measurements of pre-dawn water potential (Ψ_PD_) was performed in one fully developed leaf per plant, taken from the basal part of the primary branch with a Scholander-type pressure chamber (PMS Instruments Co., Corvallis, OR, USA) according to [35]. Measurements of Ψ_PD_ were recorded between 04:00 and 06:00 solar time. 

Additionally, the leaf relative water content (RWC) was determined according to [36] on three upper fully developed leaves, near those used to gas exchange measurements, from each genotype and treatment. RWC was calculated as Equation (1)

(1) $$\mathrm{RWC}=\frac{\mathrm{Fresh}\;\mathrm{weight}-\mathrm{Dry}\;\mathrm{weight}}{\mathrm{Saturated}\;\mathrm{weight}-\mathrm{Dry}\;\mathrm{weight}}\times 100$$

Fresh weight was determined immediately after sampling leaves. Saturated weight was obtained after incubating leaves in distilled water for 24 h under darkness at 4 °C, and dry weight was obtained after 24 h in a drying oven at 80 °C. 

### 2.4. Leaf Gas Exchange and Chlorophyll Fluorescence 

Before the beginning of the experiment, five fully developed leaves per plant were selected and marked for gas exchange and fluorescence measurements (15 independent biological replicates). Measurements were recorded for each treatment groups and after the recovery period for water-stressed plants, between 09:00 and 11:00 a.m. 

Net photosynthesis (*P_net_*), stomatal conductance (*g_s_*), and transpiration (E) were registered using an infrared gas analyzer LCPRO-SD (ADC, BioScientific Ltd., Hoddesdon, UK) associated with a red-blue LED light source on the leaf chamber. A CO_2_ cartridge was used to obtain a constant CO_2_ value at 380 µmol·mol^−1^. In the broad chamber, measures were recorded at 1400 µmol·m^−2^·s^−1^ photosynthetically active radiation, while air flow was set at 200 µmol·s^−1^, and ambient leaf temperature. When steady-state conditions were reached, data were registered (3–6 min). 

The chlorophyll fluorescence was measured on dark- and light-adapted leaves using an OS1p chlorophyll fluorimeter (Opti-Sciences, Inc. Hudson, United States). The maximum (*F*_v_/*F*_m_) quantum efficiency of PSII was recorded on dark-adapted (30 min) leaves with leaf clips after applying a saturating light pulse [37]. Non-photochemical quenching (NPQ) was calculated as NPQ = [(*F_m_* − *F*′*_m_*)/*F*′*_m_*] [38].

### 2.5. Oxidative Stress Indicators 

Three independent leaf samples (*n* = 3) for each genotype and for each treatment were harvested for biochemical assays. 

The degree of lipid peroxidation was evaluated as malondialdehyde (MDA) content in leaf samples according to [39] and adapted to citrus samples as described by [9]. A total of 80 mg powered leaf tissue was homogenized in 2 mL 80% ethanol (*v*/*v*) and centrifuged at 3000× *g* for 10 min at 4 °C. Absorbance was read at 440, 535, and 600 nm against blank. 

Hydrogen peroxide content was estimated using a PeroxiDetect Kit (Sigma, Aldrich, St. Louis, MO, USA) according to [40] and adapted to citrus samples as described by [34]. A total of 150 mg powered leaf tissue was homogenized in 300 µL of distilled water and centrifuged at 21,000× *g* for 15 min at 4 °C. Absorbance was read at 560 nm with a microplate reader (MULTISKAN FC^TM^, Thermo Scientific, Waltham, MA, USA).

### 2.6. Antioxidant Enzymatic Activities, Ascorbic Acid, and Proline Content

For extraction of the antioxidant enzymes, a total of 54 mg powered leaf tissue was homogenized in 2 mL of extraction buffer and centrifuged at 13,000× *g* for 30 min at 4 °C. The supernatant was used for CAT, APX, SOD, and DHAR activity determination, as described by [9]. Time-course measurements were recorded using a V-630 spectrophotometer (Jasco Inc., Tokyo, Japan).

Ascorbic acid content was determined following the method described by [41] and adapted to citrus samples by [34]. A total of 150 mg powered leaf tissue was homogenized in 600 µL of 6% ice-cold trichloroacetic acid (*w*/*v*) and centrifuged at 13,000× *g* for 15 min at 4°C. Absorbance was read at 550 nm with a microplate reader (MULTISKAN FC^TM^, Thermo Scientific, Waltham, MA, USA).

Free proline content was determined by using the method proposed by [42] and adapted to citrus samples by [34]. A total of 40 mg powered leaf tissue was homogenized in 70% ethanol (*v*/*v*) and centrifuged at 15,000× *g* for 15 min at 4 °C. The absorbance was read at 520 nm with a microplate reader (MULTISKAN FC^TM^, Thermo Scientific, Waltham, MA, USA). 

### 2.7. Statistical Analysis 

Statistical analyses were performed using R statistical software (http://www.R-project.org, accessed on 12 September 2020) and Rstudio (Rstudio Inc., Boston, MA, USA, version 1.4.1717). All data were expressed as means values (±standard error). The qualitative factors studied were ploidy level and treatments (control, water deficit, recovery). The variance homogeneity was checked using the Levene test, and the collected data were submitted to two-way ANOVAs followed by LSD test when a significant difference was detected. Statistical significance was set at *p* < 0.05. The normalized data were also analyzed using the multivariate analysis method based on principal component analysis (PCA) and hierarchical clustering classification (Ward’s method), using FactomineR package.

## 3. Results

Water deficit was conducted on 2x and 3x genotypes in greenhouse conditions. Plants in the control group were fully irrigated near to pot capacity, while those in the water-stressed group were maintained with manual watering at 45–55% of pot capacity over six days, wherein the physiological measurements and sampling for biochemical analysis were performed. Then, irrigation at pot capacity was restored in stressed plants for five days, and physiological data and sampling for biochemical assays were carried out to evaluate the recovery capacity.

### 3.1. Plant Water Status

**Figure 1:**
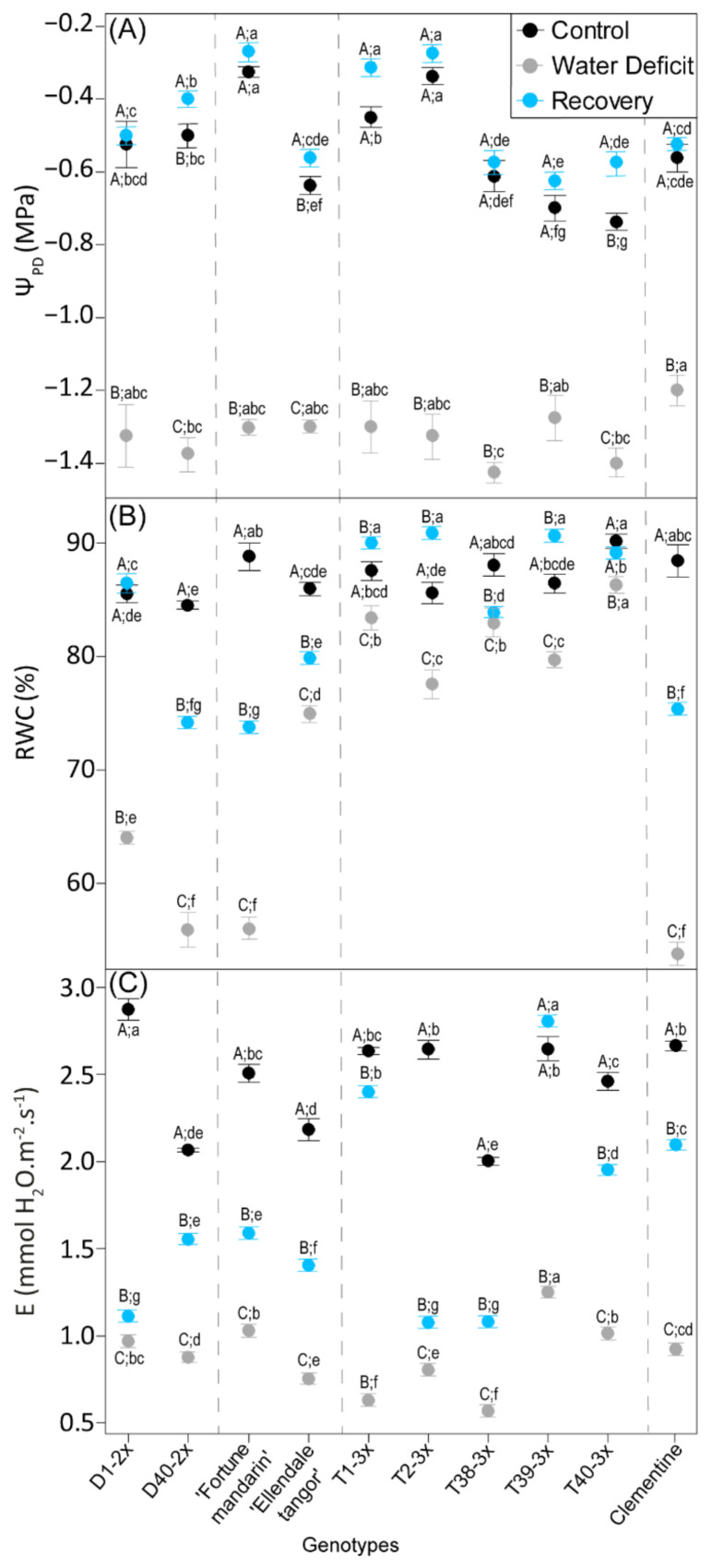
Plant water status parameters of 2x and 3x genotypes under three water treatments: 70–100% pot capacity (black point, control), 45–55% pot capacity (grey points, water deficit), and five days after re-watering (blue point, recovery). (**A**) Pre-dawn water potential (*Ψ_PD_*), (**B**) relative water content (*RWC*), and (**C**) transpiration rate (*E*). All data are mean values (± S.E.) of 3 independent measurements for Ψ_PD_ (*n* = 3) and 15 independent measurements for RWC and *E* (*n* = 15). Data were analyzed with ANOVA and Fisher’s LSD test (*p* < 0.05). Uppercase letters compare water treatments for each genotype. Lowercase letters compare genotype within the same water treatment.

For all genotypes, water deficit resulted in significant decline in plant water status parameters (Figure 1). Genotypes presented more negative values for Ψ_PD_ and lower RWC relative to control. Lowest values of RWC were found in D1-2x, Fortune mandarin, and clementine under water deficit conditions (Figure 1B). Water deficit also induced a sharp decrease in transpiration rate (E), and most of the 3x genotypes with Ellendale tangor exhibited the lowest values (Figure 1C). After the re-watering, water-related parameters increased in all genotypes. While enhanced values of RWC were obtained in 3x genotypes (between 82% and 90%), 2x genotypes, Fortune mandarin, and clementine still exhibited lower values. Despite the increase in E level after the re-watering, lower values were found in some 3x (T2-3x and T38-3x) and 2x (D1-2x, D40-2x, Fortune mandarin, and Ellendale tangor) genotypes.

### 3.2. Leaf Gas Exchange and Photochemistry Parameters

**Figure 2:**
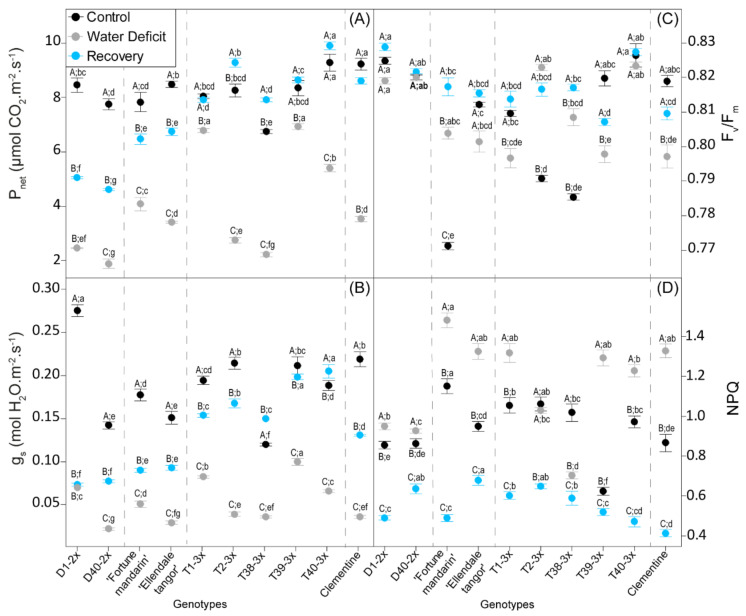
Leaf physiological parameters of 2x and 3x genotypes under three water treatments: 70–100% pot capacity (black point, control), 45–55% pot capacity (grey points, water deficit), and five days after re-watering (blue point, recovery). (**A**) Net photosynthesis (*P_net_*), (**B**) stomatal conductance (*g_s_*), (**C**) maximal quantum yield of PSII (*F_v_/F_m_*), and (**D**) non-photochemical quenching (NPQ) rate. All data are mean values (±S.E.) of 15 independent measurements (*n* = 15). Data were analyzed with ANOVA and Fisher’s LSD test (*p* < 0.05). Uppercase letters compare water treatments for each genotype. Lowercase letters compare genotype within the same water treatment.

Leaf gas exchange in terms of stomatal conductance (*g_s_*) and photosynthesis (*P_net_*) were significantly affected by water deficit (Figure 2A,B). A sharp decline of *g_s_* and *P_net_* were observed in all genotypes, although the highest values were recorded in most 3x genotypes. After re-watering, the leaf gas exchange recovered in all genotypes, and 3x genotypes still exhibited the greatest values. 

Chlorophyll fluorescence was analyzed in terms of maximum efficiency of PSII photochemistry (*F_v_/F_m_*) and non-photochemical quenching (NPQ) (Figure 2C,D). Water deficit as re-watering induced slight modifications in *F_v_/F_m_* ratio since it was close to the physiological value (≈0.83). NPQ values rise significantly in all genotypes, except for T2-3x, where values were similar to the control, and T38-3x, which exhibited a decline of NPQ. Overall, most of the 3x genotypes and both parents showed the greatest NPQ values during water deficit. After re-watering, values of NPQ declined in all genotypes, where slight significant differences were observed between the genotypes.

### 3.3. Oxidative Damages and Antioxidant Response

**Figure 3:**
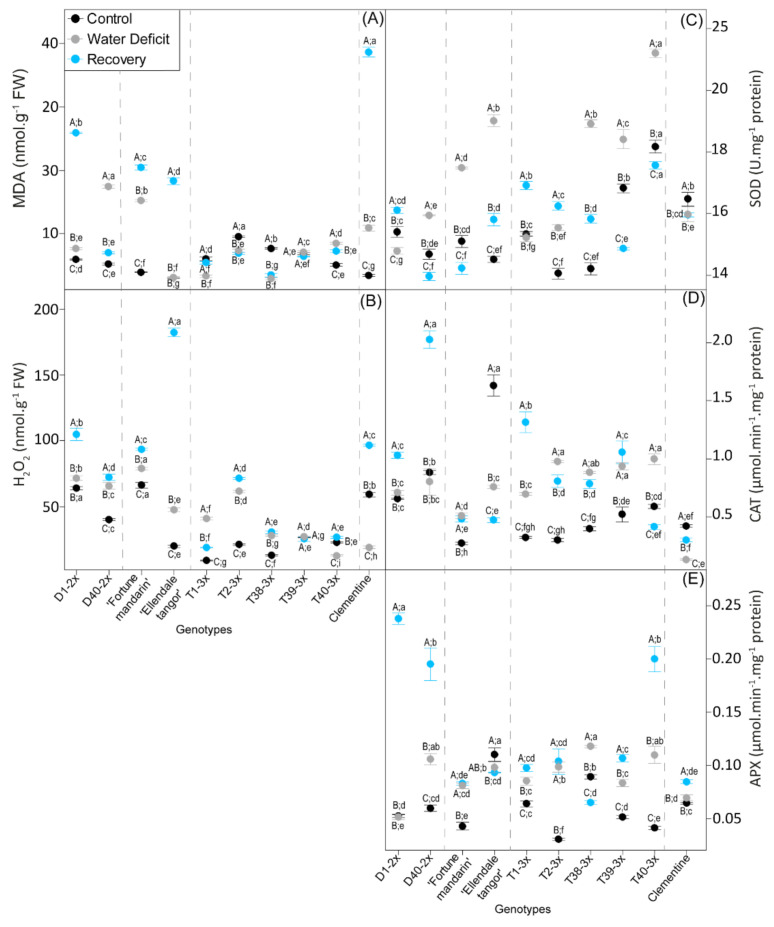
Oxidative stress markers and antioxidant enzymes in 2x and 3x genotypes under three water treatments: 70–100% pot capacity (black point, control), 45–55% pot capacity (grey points, water deficit), and five days after re-watering (blue point, recovery). (**A**) Malondialdehyde (MDA); (**B**) H_2_O_2_ content; (**C**) specific activities of SOD, (**D**) CAT, and (**E**) APX. All data are mean values (±S.E.) of 15 independent measurements (*n* = 15). Data were analyzed with ANOVA and Fisher’s LSD test (*p* < 0.05). Uppercase letters compare water treatments for each genotype. Lowercase letters compare genotype within the same water treatment.

Oxidative damages were evaluated through H_2_O_2_ and MDA accumulation (Figure 3A,B). Both water deficit and recovery significantly increased H_2_O_2_ and MDA accumulation in most of the genotypes. No significant difference of both oxidative stress markers was observed in T39-3x compared with control values. Overall, 2x genotypes showed the highest values of MDA and H_2_O_2_ accumulation under water deficit. After re-watering, 3x genotypes exhibited the lowest values of oxidative stress markers. 

In response to water deficit, stimulation of antioxidant enzymatic defenses, in terms of SOD, CAT, and APX, were observed in most of genotypes (Figure 3C–E). The highest activity of SOD was found in some 3x genotypes (T38-3x, T39-3x) and both parents (Figure 3C). While no significant variation of CAT activity was observed between water-stressed and control plants in 2x genotypes, the greatest values were found in most 3x genotypes (Figure 3D). T38-3x and T40-3x exhibited the highest values of APX activity (Figure 3E). 

After re-watering, decrease in SOD activity was observed in most of the genotypes. Triploid genotypes exhibited higher values in comparison with 2x genotypes. A rise of CAT and APX activities were also observed after the recovery period in some 2x and 3x genotypes. After re-watering, 2x genotypes exhibited the greatest values of CAT and APX activities. 

**Figure 4:**
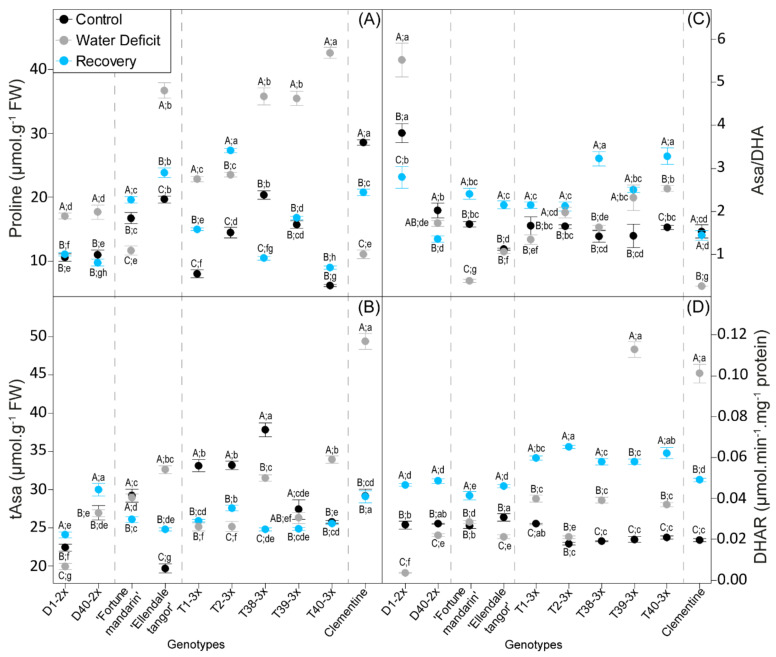
Changes in (**A**) proline, (**B**) tAsa content, (**C**) Asa/DHA ratio, and (**D**) specific activity of dehydroascorbate reductase (DHAR) in 2x and 3x genotypes under three water treatments: 70–100% pot capacity (black point, control), 45–55% pot capacity (grey points, water deficit), and five days after re-watering (blue point, recovery). All data are mean values (±S.E.) of 15 independent measurements (*n* = 15). Data were analyzed with ANOVA and Fisher’s LSD test (*p* < 0.05). Uppercase letters compare water treatments for each genotype. Lowercase letters compare genotype within the same water treatment.

Antioxidant defenses in terms of proline and ascorbate redox status were evaluated in selected genotypes in response to water deficit (Figure 4). Water deficit induced a rise in proline content except in Fortune mandarin and clementine (Figure 4A). Triploid genotypes showed higher values in comparison with diploid ones. In response to water deficit, a decrease in tAsa was observed in all genotypes, except in T40-3x and clementine, which exhibited the greatest value (Figure 4B). Slight changes in Asa/DHA ratio were reported in most of genotypes between stressed plants and control ones (Figure 4C). Fortune mandarin and clementine exhibited the lowest values of Asa//DHA ratio during water deficit. Increase in DHAR activity was reported in response to water deficit in 3x genotypes and clementine, while a decline was observed in 2x genotypes (Figure 4D). The greatest DHAR activities were found in T39-3x and clementine during water deficit.

After the re-watering, a sharp drop in proline content was reported in most of genotypes, while Fortune mandarin, clementine, and T2-3x showed an increase in response to rehydration. Slight significant differences of tAsa content were observed between genotypes at the recovery period, while an increase in Asa/DHA ratio was reported in most of genotypes, and highest values were found in T38-3x and T40-3x. A sharp rise of DHAR activity was observed after re-watering in all genotypes (except T39-3x and clementine), and the highest value was found in the T2-3x genotype.

### 3.4. Overall Assessment of the Physiological and Biochemical Processes during Water Deficit and after Re-Watering 

**Figure 5:**
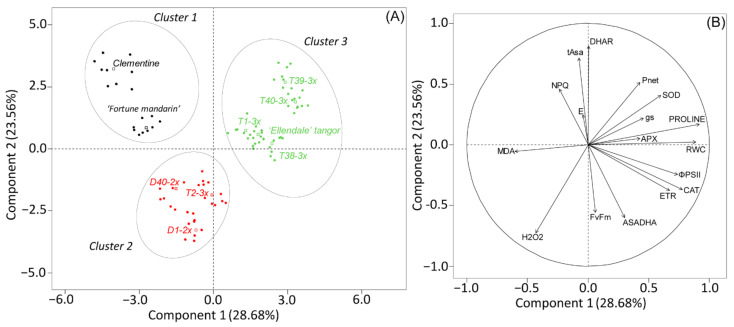
Principal component analysis (PCA) performed on leaves of 2x and 3x genotypes subjected to water deficit. (**A**) Clustering of genotypes on the two first components and (**B**) contribution of each physiological and biochemical parameter to the two first components of PCA.

Physiological and biochemical parameters monitored under water deficit and after the re-watering were subjected to PCA. Figure 5 depicted the response to water deficit where the two first components explained 52.21% of the total variability. Proline and RWC were positively correlated with component 1, and only MDA was negatively correlated with component 1. Protective mechanisms in terms of tAsa, DHAR, and NPQ were positively correlated with component 2, while H_2_O_2_, *F_v_/F_m_*, and Asa/DHA were negatively correlated with component 2. The discriminant analysis characterized the genotypes in three distinct clusters (1–3). Cluster 1 (Clementine and Fortune mandarin) and cluster 2 (diploid genotypes and T2-3x) were differentiated from cluster 3 (3x genotypes and Ellendale tangor) by lower proline and RWC content and enhanced oxidative damages (MDA and H_2_O_2_ content). Cluster 2 was also characterized by less protective mechanisms (NPQ, tAsa content, and DHAR activity) in comparison with Cluster 1.

**Figure 6:**
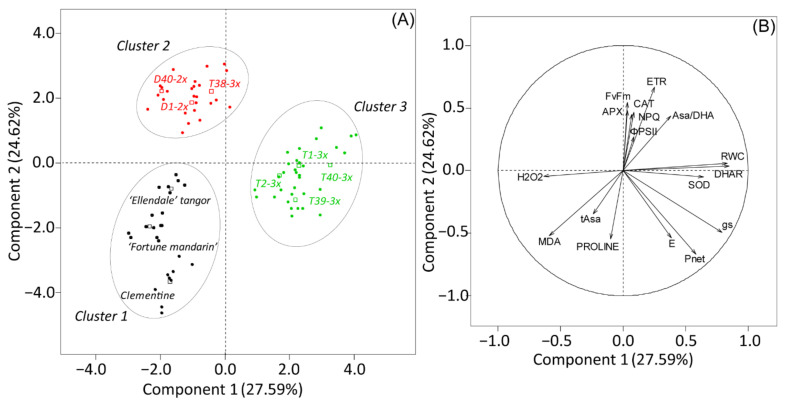
Principal component analysis (PCA) performed on leaves of 2x and 3x genotypes after the recovery period. (**A**) Clustering of genotypes on the two first components and (**B**) contribution of each physiological and biochemical parameter to the two first components of PCA.

Figure 6 depicted the response to recovery where the two first components explained 56.24% of the total variability. Component 1 was positively correlated with antioxidant enzymes (SOD, DHAR) and RWC and negatively correlated with H_2_O_2_. Component 2 was positively correlated with chlorophyll fluorescence related parameters (*F_v_/F_m_*, NPQ) and antioxidant enzymatic defenses (APX, CAT), while it was negatively correlated with physiological (*P_net_*, *E*) and biochemical (tAsa, proline) parameters. Discriminant analysis characterized the genotypes into three distinct clusters (1–3). Cluster 3 (triploid genotypes) was differentiated from clusters 1 (clementine and both parents) and 2 (2x genotypes, T38-3x genotype) by enhanced RWC and antioxidant enzymatic mechanism after re-watering with less oxidative marker accumulation. Cluster 1 was also differentiated from other clusters by higher antioxidant components (tAsa and proline), while physiological parameters remained weak after re-watering, in comparison with cluster 2.

## 4. Discussion

Drought is one of the main environmental stressors limiting crop production, due to restricting water availability. As expected, water deficit applied in our experiment led to a sharp decline of water status-related parameters (Figure 1). Pre-dawn water potential, a key indicator of soil water status, declined significantly in stressed plants, indicating that water deficit occurred in 2x and 3x genotypes. As the values of *Ψ_PD_* ranged between −1.2 MPa and −1.4 MPa, we may consider that all genotypes were subjected to moderate water deficit [43]. Since stomatal closure occurs at *Ψ_PD_* near to −1 MPa and −2 MPa [44], these conditions are enough to induce stomatal closure. Due to water unavailability in water deficit, plant metabolism and physiological processes may thus be significantly hampered.

### 4.1. Are Triploid Genotypes More Tolerant or Resilient to Water Deficit?

Photosynthesis is one of the most sensitive processes to water deficit [45]. In our study, a greater ability to sustain an enhanced photosynthetic capacity was observed in 3x genotypes, as revealed by greater *P_net_* and *g_s_* values during water deficit (Figure 2 and Figure 5). Some studies reported the ability of polyploid to sustain an effective photosynthetic apparatus and water use in response to drought [46,47]. Such trends were also observed in polyploid citrus under severe water deficit [31,48] or salinity stress [29]. Moreover, enhanced leaf gas exchange parameters were reported in 3x Persian Lime subjected to HLB stress [32]. These enhanced photosynthetic capacity was also related to morphological properties induced by polyploidy [27,49]. Indeed, higher stomatal conductance can be found in polyploids and resulted from greater stomatal opening [50]. Moreover, the greater instability of triploid plants caused by their unbalanced chromosomal set may allow a large variation in gene expression [27]. Hence, [51] reported that 3x accession of *Lippia alba* showed higher expression of photosynthesis-related proteins, in comparison with 2x ones, which can explain the greater photosynthetic capacity under environmental stresses. 

Sustaining photosynthesis rate under water deficit is crucial to limit increased light energy absorption and over-reduction of photosynthetic electron chain, resulting in photo-oxidative stress by ROS accumulation. In addition, H_2_O_2_ accumulation can result in severe cell damages, such as membrane lipid peroxidation through hydroxyl radical production [52]. In the present study, 3x genotypes showed less accumulation in H_2_O_2_ and MDA content during water deficit in comparison with 2x genotypes (Figure 3A,B and Figure 5). This lower level of oxidative damage could have been due to an efficient antioxidant defense system in the 3x genotypes. Less lipid peroxidation in response to environmental stresses was also reported in both 3x scions [31] and tetraploid rootstocks [53], and was associated with improved tolerance to oxidative stress. According to the cluster repartition, we can suggest that 3x genotypes were more tolerant to moderate water deficit than 2x genotypes. 

The ability to sustain a stronger photosynthetic capacity and less oxidative damages during drought has usually been associated with a fast recovery after re-watering [54]. In our study, PCA performed after the recovery period separated 3x genotypes from 2x genotypes according to their recovery capacity (Figure 6). Triploid genotypes, gathered in the cluster 3 (except T38-3x genotype), fully recovered photosynthetic capacity, as suggested by increased leaf gas exchange (Figure 2A,B). These genotypes also exhibited a better recovery in terms of water status parameters (Figure 6), although lower E values were found in some 3x genotypes (Figure 1C). Despite the increase in leaf gas exchange parameters in response to re-watering, 2x genotypes still exhibited lower values, in comparison with 3x genotypes. The very low stomatal conductance values found in 2x genotypes may indicate that *g_s_* had a partial recovery of stomatal aperture after re-watering. As H_2_O_2_ is related to stomatal closure signaling [55], we can hypothesize that the weak *g_s_* were related in H_2_O_2_-induced control of stomatal closure, also following the rise in water availability. Flexas et al. [56] further indicated that stomatal and mesophyll conductance limitations would be responsible for the slow recovery after re-watering. 

Both cluster 1 and 2, containing 2x genotypes, exhibited increased H_2_O_2_ accumulation after the recovery period (Figure 6), and could have been due to acceleration of the Haber–Weiss reaction that leads to hydroxyl radical formation [6] and consequently to MDA accumulation (Figure 3A and Figure 6). Thus, oxidative stress persisted in 2x genotypes, despite plant rehydration that may have been due to inefficient repair processes of the photosynthetic apparatus. In contrast, oxidative marker accumulation was effectively managed in most of the 3x genotypes after re-watering (Figure 6), which could also have been due the low accumulation during water deficit. These results suggested that reducing damages of photosynthetic system during water deficit can contribute to rapid recovery of plants. The same conclusions have been reported in tetraploid plants of *Lonicera Japonica* and triploid *Populus* under drought stress [57,58]. 

Taken together, our results indicated that 3x genotypes were more tolerant and resilient to water deficit than 2x ones through their ability to preserve the stability of the photosynthetic system and to sustain leaf water status, which may be due to efficient protective mechanisms.

### 4.2. Does Osmotic Adjustment Explain the Better Sustain of Leaf Water Status in Triploid Genotypes?

Decrease in RWC, the main indicator of leaf water status, is the earliest effect of water deficit in plants [59,60]. As expected, water deficit conditions led to low RWC and transpiration rate (Figure 1B,C). The decline of water status in response to drought has been previously reported in citrus [61,62] and other species such as *Maclura pomifera* [63] or *Jatropha curcas* L. [64]. It is well known that RWC is not directly related to the leaf osmotic adjustment (OA), but it expresses the effect of OA [65]. Thus, maintaining well water status may be associated to effective osmotic adjustment in the leaf. This mechanism induces accumulation of compatible solutes, or osmolytes, such as proline, that was widely associated with drought tolerance [29,66]. In our study, triploid genotypes showed a greater RWC and proline accumulation during water deprivation, in comparison with 2x genotypes (Figure 5). These results indicate a better water use strategy through osmotic adjustment under water deficit in 3x genotypes. Our findings were in agreement with the study of [57], which showed that honeysuckle tetraploid cultivars exhibited greater resistance to water stress than diploids in terms of osmolyte compounds and leaf gas exchange. Such trends in maintaining water balance have also been observed in polyploid citrus, including tetraploid [48] and 3x genotypes [31]. Overall, 3x genotypes showed an effective osmotic adjustment to maintain high RWC during water deficit, which may limit photo-inhibition.

### 4.3. Does Non-Photochemical Quenching Explain the Lower Oxidative Damage of the Photosynthetic Apparatus?

Non-photochemical quenching plays a crucial role in leaf photoprotection under environmental stresses such as water deficit because it can dissipate more than 75% of the photons absorbed by the leaves [67]. In our study, increased NPQ (Figure 2D) was observed in all genotypes and could explain the slight variation of maximal photochemical efficiency (*F_v_/F_m_*), which reached values mostly obtained under optimal conditions (≈0.83), regardless the genotype or the ploidy level (Figure 2C). These results indicated that photo-protective mechanisms were efficient to prevent photo-inhibition under water deficit. Although the greater NPQ values were found in 3x genotypes (Figure 2D), we cannot discriminate 2x from 3x in terms of chlorophyll fluorescence. Furthermore, rise in NPQ may be related to oxidative damages induced by ROS accumulation, through the antioxidant role of xanthophyll cycle, involving ascorbate as electron donor [14]. In our study, a positive correlation was observed between ascorbate content and NPQ observed for cluster 1 (clementine and Fortune mandarin) and cluster 3 (3x genotypes and Ellendale tangor) under water deficit (Figure 5) that could indicate an effective stimulation of protective mechanism to stabilize thylakoid membranes under water deficit. Hence, enhanced NPQ with upregulation of xanthophyll cycle has already been reported in polyploids under light stress [68] or severe water deficit [31], and was associated with the limiting of oxidative damage.

### 4.4. Does the Antioxidant Defence System Explain the Better Tolerance to Water Deficit in Triploid Genotypes?

Under stressful conditions, antioxidant processes play a crucial role in maintaining a balance between ROS accumulation and detoxification in order to limit oxidative stress-related damages. In our study, increased specific SOD activity found in 3x genotypes and Ellendale tangor was related to low MDA levels and thus the limitation of the lipid peroxidation process (Figure 3A). In contrast, rise in SOD activity was also reported in Fortune mandarin but appeared insufficient to limit MDA accumulation during water deficit (Figure 3 and Figure 5). CAT and APX both help to limit H_2_O_2_ accumulation in various cell compartments [19]. Increased APX and CAT activities reported in most of the 3x genotypes appeared sufficient to prevent oxidative damage by reducing H_2_O_2_ accumulation (Figure 3B). Increased SOD and APX activities were also observed in clementine grafted onto tetraploid Carrizo citrange under natural chilling stress [69]. In the same way, stronger APX and CAT activities were recorded in triploid Persian lime, which favored the oxidative stress-induced HLB limitation [32]. Moreover, these authors reported that CAT activity was the key marker that differentiate the triploid Persian lime from 2x Mexican lime under HLB stress. 

Moreover, increased APX activity could imply the involvement of the Halliwell–Asada pathway to limit ROS accumulation. Ascorbate is one of the powerful antioxidant molecule protecting cell from oxidative damage imposed by abiotic stresses, including water deficit [6]. DHAR, which is involved in the Halliwell–Asada pathway, can regenerate Asa from the oxidized form and thus regulate the cellular ascorbate redox state. In our study, better DHAR activity (Figure 4D), mostly in 3x genotypes, was associated with a constant or an increase Asa/DHA ratio above 1 (Figure 4C). This result indicated that DHAR activity was enough for an effective recycling of Asa and contributed to stronger antioxidant capacity. In contrast, clementine and Fortune mandarin showed an Asa/DHA ratio below 1, despite the rise in DHAR activity and the enhanced de novo ascorbate synthesis (only in clementine) in response to water deficit, which implies an impaired recycling process of Asa in these genotypes. 

In addition to its role as an osmoprotectant, proline is also known as an effective scavenger of singlet oxygen and radical hydroxyl, preventing lipid peroxidation during environmental stresses [12,70]. In our study, higher proline accumulation in 3x genotypes and Ellendale tangor (Figure 4A and Figure 5) with less oxidative marker accumulation (Figure 3) suggested an effective control of cell redox status and a better preservation of the integrity of thylakoid membranes during water deficit. High proline was already reported in 3x *Populus* subjected to drought [58] and salt stress [22], as well as tetraploid citrus seedlings under salinity stress [29]. Moreover, proline accumulation observed in 3x citrus varieties was related to less chloroplast damage under severe water deficit [49]. Taken together, these results revealed that the enhanced antioxidant system found in 3x genotypes was efficient in controlling oxidative marker accumulation and thus limiting cell damage. Previous studies also reported that polyploids showed enhanced antioxidant system defenses that were related to a better tolerance to abiotic stresses including water deficit [31,47,71]. 

Increasing importance has been given to a plant’s ability for water deficit recovery, especially for crops [45]. Our PCA performed at recovery period clearly discriminated 3x genotypes from 2x ones, mostly in terms of antioxidant defenses (Figure 6). While SOD activity declined with increased RWC, increased APX, CAT, and DHAR activities were observed in most of the genotypes (Figure 3). This result may indicate a later response of the antioxidant system to water deficit. In 3x genotypes, these rises were associated with effective control of MDA and H_2_O_2_ accumulation (Figure 3). In contrast, increase in oxidative marker level was associated to 2x genotypes, and enhanced antioxidant enzymatic activities were not enough to limit them. We can also hypothesize that enhanced oxidative markers during recovery may result in impairment of the photosynthetic apparatus, as suggested by partial recovery of leaf gas exchange parameters (Figure 2). These results revealed that triploidy can reduce oxidative damage during water deficit, which contributes to a full recovery. Diploid genotypes still suffered oxidative stress even after a water deficit episode. Munne-Bosh et al. [5] also reported that oxidative stress can be more severe during the recovery phase when the RWC drops sharply during water deficit. Our findings agree with previous studies in which polyploids with increased stress tolerance also displayed an enhanced antioxidant mechanism [29,31,53]. 

Overall, it is interesting to note that the enhanced genetic diversity and buffering effects of gene redundancy observed in polyploid lines were related to a better tolerance to environmental stresses [72,73]. Indeed, enhanced transcript expression of antioxidant enzymes genes (SOD, CAT, and APX) has been reported in tetraploids under drought conditions [74]. Moreover, several genes encoding transcription factors including DREB, MYB, NAC, or WRKY were induced specifically or to a greater level in tetraploid lines under cold, drought, and salt stress [23,71].

## 5. Conclusions

In this study, we found that triploid showed a better tolerance to water deficit in terms of water status-related parameters, leaf gas exchange, and antioxidant defense mechanisms, in comparison with diploid genotypes. Hence, effective osmotic adjustment and antioxidant enzymatic activities may explain the lower oxidative damage and sustain photosynthetic capacity during water deficit in 3x genotypes. We also found that triploidy clearly improved the recovery capacity of photosynthetic activity and biochemical processes after a water deficit episode, which may have been to the lower level of damage to the photosynthetic apparatus during the stress. The ability to resist to water deficit but also to restore physiological and biochemical processes appeared to be a key advantage for crop sustainability under increased drought conditions suggested by climate change predictions. Finally, our study provides a framework for fundamental biochemical analyses that can be used to discriminate water deficit-tolerant and -sensitive genotypes among a citrus breeding program to select interesting cultivars. To confirm the better water deficit response of these 3x genotypes, field experimentations will be conducted on adult trees. Associated with genetic studies, this knowledge is of great value for plant breeding programs aimed at developing new citrus varieties under stress conditions. Since the better water deficit response may be due to differential gene expression resulting from hybridization and/or polyploidy per se, future investigations should focus on the molecular determinants of the better water deficit tolerance of triploid genotypes.

## Data Availability

Data is contained within the article.
